# Reframing Patient-Centered Medical Education

**DOI:** 10.1212/NE9.0000000000200367

**Published:** 2026-09-18

**Authors:** Roy E. Strowd

**Affiliations:** Wake Forest University, Winston-Salem, NC.

## Patient Outcomes as the Ultimate Educational Outcome

Medical education has never existed simply to create knowledgeable clinicians. Every lecture, bedside discussion, simulation, and feedback conversation ultimately shares a common purpose: improving the lives of patients. Patients are, and always have been, the ultimate beneficiaries of medical education.

Yet, patient outcomes remain surprisingly uncommon end points in medical education research. Education innovations are designed to improve patient care, yet are typically judged by learner knowledge, confidence, skills, or milestones rather than whether patients ultimately benefit. These educational outcomes are essential, but are mere pit stops on the path to the outcome we ultimately seek, better patient care.

This paradox raises an important question. If the purpose of medical education is to improve patient care, should patient outcomes increasingly become the ideal outcome for education research?

The answer is not as straightforward as it first appears. Patient outcomes have traditionally been viewed as the domain of clinical quality improvement, implementation science, or health services research rather than education. Yet this distinction deserves reconsideration.

## Educational Research and Quality Improvement: More Similar Than We Think

Education research and clinical quality improvement are often viewed as distinct disciplines. In reality, both seek to improve patient care.

The defining difference is not the outcome they seek but the intervention they use. Quality improvement intervenes directly within health care delivery through changes in clinical workflows, operational processes, care pathways, or health systems. Education research intervenes upstream by changing the knowledge, skills, behaviors, attitudes, reasoning, and professional judgment of clinicians who subsequently care for patients. The mechanism differs, but the destination remains remarkably similar (Table).

**Table:** Comparison of Clinical Quality Improvement and Educational Research as Complementary Approaches to Improving Patient Care

	Clinical quality improvement	Educational research
Intervention	Workflow redesign, care pathway, operational change, decision support	Curriculum, simulation, coaching, assessment, feedback, faculty development
Primary target	Healthcare systems	Health care professionals and trainees
Mechanisms	Process redesign	Learning and behavior change
Immediate outcomes	Efficiency, reliability, adherence, safety	Knowledge, skills, clinical reasoning, confidence, behaviors, entrustment
Ultimate outcome	Better patient care	Better patient care

Educational research and quality improvement should not be viewed as competing approaches to improving patient care. They are complementary sciences that intervene at different points along the same pathway.

Recognizing this distinction changes how we think about educational scholarship. Patient outcomes should not be viewed as belonging exclusively to quality improvement. They represent the shared aspiration of both fields.

## Patient-Centered Medical Education

Patient-centered care transformed medicine by redefining success around outcomes that matter most to patients. Medical education should undergo a similar transformation.

Patient-centered medical education begins by understanding educational needs. Just as effective patient care begins with an accurate diagnosis, effective education begins with understanding the clinical realities learners face. Two studies in this issue of *Neurology*® *Education* illustrate complementary approaches to this challenge. One uses electronic health record data to characterize the patient populations residents actually encounter, revealing important gaps between authentic clinical experience and expected educational content.^1^ Another explores the roots of neurophobia through qualitative inquiry, demonstrating that educational barriers arise not only from difficult content but also from learner perceptions, curricular structure, and the black box nature of neurology itself.^2^ Together, these studies remind us that meaningful education innovation begins with understanding both learners and the patients they serve.

## Measuring What Matters

Once educational needs are understood, the challenge is designing interventions that meaningfully change clinical practice. Not every educational study should measure patient outcomes. Early investigations appropriately establish feasibility and learner outcomes before examining whether educational gains translate into clinical practice.

Most education innovations appropriately begin by establishing the impact on learners. The educational innovations in this issue are remarkably diverse, ranging from simulation^3^ and workplace-based assessment^4^ to artificial intelligence,^5^ and targeted disease-specific curricula.^6^ Although their instructional methods differ considerably, each shares the same objective: improving clinician performance so that future patients receive better care.

## A Call to the Field

Clinical medicine has long recognized the distinction between surrogate end points and clinical outcomes. Blood pressure predicts stroke. Glycated hemoglobin predicts diabetic complications. Tumor response predicts survival. These measures are invaluable because they provide early evidence that therapies are working, but they are rarely the outcomes patients ultimately value.

Medical education has its own surrogate end points. Knowledge, confidence, entrustment, and certification performance function as educational biomarkers. They provide indispensable early evidence that an intervention is working. Their value lies not simply in what they measure today, but in what they predict about tomorrow's patient care.

The next frontier for educational science is not simply demonstrating that learning occurred but demonstrating that learning mattered. Knowledge is valuable only if it changes clinical behavior. Educational interventions ultimately fulfill their purpose when those behavioral changes translate into better care for patients.

Several studies in this issue begin to explore this critical transition. The cross-disciplinary simulation curriculum found that most residents subsequently encountered patients resembling simulated scenarios and reported greater confidence in counseling families and interpreting clinical findings months later.^7^ This moves beyond immediate learner reactions toward the transfer of learning into authentic clinical environments. Similarly, EEG workplace-based assessments documented progressive reductions in faculty assistance over a two-week elective, providing evidence that learners were becoming increasingly capable of performing clinical work with less supervision.^4^

The investigators evaluating the LouiS artificial intelligence platform explicitly recognize this evolution.^5^ They appropriately note that improved case-based performance represents only one step in the educational pathway and identifies knowledge retention, behavior change, and real-world clinical outcomes as the logical next questions. This reflects an encouraging maturation of educational science from asking whether learning occurred to asking whether learning ultimately changes patient care.

Several forces make this an opportune time to reconsider how we evaluate educational effectiveness. Electronic health record data increasingly allow investigators to connect educational experiences with authentic clinical practice. Artificial intelligence can facilitate longitudinal assessment of clinical reasoning, decision making, and documentation at a scale previously impossible. Competency-based medical education has shifted attention toward clinical performance. Together, these advances create new opportunities to examine educational outcomes farther downstream than previously feasible.

This is not a call to abandon learner outcomes or to expect every educational innovation to measure patient outcomes. Early studies should establish educational effectiveness; mature interventions should increasingly examine how learning influences clinician behavior, health care delivery, and patient care.

## Conclusion

Collectively, the studies in this issue illustrate a field progressing from understanding educational needs to designing interventions, demonstrating learner outcomes, and increasingly exploring their influence on clinical practice. The next frontier is not replacing learner outcomes but extending them to demonstrate better patient care.

Patients have always been the reason we teach. Patient outcomes should increasingly become the way we judge whether our teaching succeeds. Happy reading.

## References

[1] Gottlieb-Smith R, Adams J, Bernson-Leung ME, et al. Education Research: educational informatics: characterizing child neurology resident clinical experiences using electronic health record data. Neurol Educ. 2026;5(3):e200334. doi:10.1212/NE9.0000000000200334 PMC13316738 42381653

[2] Korb PJ, McGuinness I, Hawkins T, Corral J. Education Research: explaining neurophobia in medical students: a comparison with math anxiety. Neurol Educ. 2026;5(3):e200340. doi:10.1212/NE9.0000000000200340 PMC13372129 42459924

[3] Mustafa R. A generational approach to neurology education: engaging generation Z Learners. Neurol Educ. 2026;5(3):e200355. doi:10.1212/NE9.0000000000200355 PMC13485139 42615013

[4] Kostan H, Dickey A, Gadelmola K, et al. Education Research: development of a workplace-based assessment to enhance EEG interpretation skills for medical students. Neurol Educ. 2026;5(3):e200349. doi:10.1212/NE9.0000000000200349 PMC13494825 42630615

[5] Fagundes TP, Clares De Andrade JB, Mota AMSA, Nishimura VDMR, Soga FH, Camilo M. Education Research: validation of an AI-powered educational platform for neuroanatomical localization and stroke syndrome recognition: a multicenter study. Neurol Educ. 2026;5(3):e200350. doi:10.1212/NE9.0000000000200350 PMC13485138 42615002

[6] Viola M, Reda H, Hovaguimian A, Zuflacht J. Education Research: case-based and near-peer teaching on functional neurologic disorder in the neurology clerkship: a cross-site comparative pilot study. Neurol Educ. 2026;5(3):e200342. doi:10.1212/NE9.0000000000200342 PMC13330575 42404405

[7] Nerbonne E, Magelssen S, Muhlhofer WG. The impact and benefits of cross-disciplinary educational collaboration between a department of neurology and department of drama. Neurol Educ. 2026;5(3):e200361. doi:10.1212/NE9.0000000000200361

